# Rapid Online Corrections for Proprioceptive and Visual Perturbations Recruit Similar Circuits in Primary Motor Cortex

**DOI:** 10.1523/ENEURO.0083-23.2024

**Published:** 2024-02-09

**Authors:** Kevin P. Cross, Douglas J. Cook, Stephen H. Scott

**Affiliations:** ^1^Neuroscience Center, University of North Carolina, Chapel Hill, North Carolina 27599; ^2^Department of Surgery, Queen’s University, Kingston, Ontario K7L 3N6, Canada; ^3^Centre for Neuroscience Studies, Queen’s University, Kingston, Ontario K7L 3N6, Canada; ^4^Departments of Biomedical and Molecular Sciences, Queen’s University, Kingston, Ontario K7L 3N6, Canada; ^5^Medicine, Queen’s University, Kingston, Ontario K7L 3N6, Canada

**Keywords:** motor cortex, multisensory, proprioception, vision

## Abstract

An important aspect of motor function is our ability to rapidly generate goal-directed corrections for disturbances to the limb or behavioral goal. The primary motor cortex (M1) is a key region involved in processing feedback for rapid motor corrections, yet we know little about how M1 circuits are recruited by different sources of sensory feedback to make rapid corrections. We trained two male monkeys (*Macaca mulatta*) to make goal-directed reaches and on random trials introduced different sensory errors by either jumping the visual location of the goal (goal jump), jumping the visual location of the hand (cursor jump), or applying a mechanical load to displace the hand (proprioceptive feedback). Sensory perturbations evoked a broad response in M1 with ∼73% of neurons (*n* = 257) responding to at least one of the sensory perturbations. Feedback responses were also similar as response ranges between the goal and cursor jumps were highly correlated (range of *r* = [0.91, 0.97]) as were the response ranges between the mechanical loads and the visual perturbations (range of *r* = [0.68, 0.86]). Lastly, we identified the neural subspace each perturbation response resided in and found a strong overlap between the two visual perturbations (range of overlap index, 0.73–0.89) and between the mechanical loads and visual perturbations (range of overlap index, 0.36–0.47) indicating each perturbation evoked similar structure of activity at the population level. Collectively, our results indicate rapid responses to errors from different sensory sources target similar overlapping circuits in M1.

## Significance Statement

Motor actions often require continuously integrating multiple sources of information, such as the locations of a goal and your limb. The primary motor cortex (M1) plays a critical role in correcting for unexpected errors to the goal and limb. How might goal and limb feedback recruit circuits in M1 during a motor correction? We found M1 responded similarly for errors to the visual feedback of the goal and errors to the visual feedback of the limb. Furthermore, errors to the proprioceptive feedback of the limb and visual errors evoked similar patterns of activity in M1. Collectively these results suggest a partial convergence of feedback sources in M1 that supports online control.

## Introduction

Sensory feedback plays a critical role in ensuring motor actions are successfully performed, providing information about motor errors due to external disturbances and internal noise inherent in the motor system. Sensory feedback is also essential for generating overt corrections such as when someone bumps your arm while moving or when the behavioral goal unexpectedly moves such as a glass tipping when the table is bumped. While vision plays a dominant role for identifying most behavioral goals, both vision and proprioception are available for feedback about the limb. Performing most motor actions thus requires combining visual feedback of the goal with feedback of the limb from proprioception and vision.

The primary motor cortex (M1) plays an important role in generating goal-directed corrections. M1 receives rich inputs from brain regions involved in proprioceptive and visual processing including the parietal and frontal cortices (Jones et al., 1978; Zarzecki and Strick, 1978; Crammond and Kalaska, 1989; Porter and Lemon, 1993; Buneo et al., 2002; Pesaran et al., 2006; McGuire and Sabes, 2011; Bremner and Andersen, 2012; Dea et al., 2016; Omrani et al., 2016; Gamberini et al., 2017; Piserchia et al., 2017; Kalidindi et al., 2021; Takei et al., 2021) and the cerebellum (Conrad et al., 1975; Vilis et al., 1976; Strick, 1983; Guo et al., 2020; Sauerbrei et al., 2020). M1 responds to proprioceptive feedback of the limb within ∼20–40 ms of a mechanical load (Evarts and Tanji, 1976; Wolpaw, 1980; Lemon, 1981a; Suminski et al., 2009; Pruszynski et al., 2011, 2014; Omrani et al., 2014; Heming et al., 2019) and to visual feedback of the limb and goal within ∼70 ms (Georgopoulos et al., 1983; Cisek and Kalaska, 2005; Ames et al., 2014; Stavisky et al., 2017). However, little is known about how these different sources of sensory information (i.e., vision of the goal, vision of the hand, and proprioception of the hand) recruit M1 circuits for rapid feedback corrections.

On one extreme, each feedback source may influence M1 independently to generate the motor correction (independence hypothesis). The motor system rapidly responds to proprioceptive and visual feedback, which may not allow the brain sufficient time to perform the necessary nonlinear computations. Anatomical evidence highlights how different areas involved with sensory processing appear to target spatially distinct circuits in M1. For example, somatosensory and parietal areas project to more caudal subdivisions of M1, whereas areas involved with visual processing such as premotor cortex project to more rostral subdivisions (Dea et al., 2016). Further, many studies highlight how M1 neurons that responded to cutaneous and proprioceptive inputs are distinct with only rare instances of neurons that responded to both stimuli (Rosén and Asanuma, 1972; Lemon et al., 1976; Wong et al., 1978; Fetz et al., 1980; Tanji and Wise, 1981; Strick and Preston, 1982; Picard and Smith, 1992). These studies suggest that inputs carrying sensory information to M1 may be segregated leading to recruitment of distinct circuits in M1 for proprioceptive and visual corrective motor responses with overlap only by random chance.

Alternatively, feedback sources could recruit a similar population of M1 neurons to generate the motor correction (convergence hypothesis). Integrated feedback is an assumption in many control schemes where a difference vector is computed between the location of the goal and hand, which is then converted to motor ,nds (Bullock et al., 1998; Sober and Sabes, 2003; Shadmehr and Wise, 2005; Burns and Blohm, 2010). A common assumption is the computation of the difference vector occurs in premotor and parietal cortices where neural signatures of this computation have been found (Buneo et al., 2002; Pesaran et al., 2006; McGuire and Sabes, 2011; Bremner and Andersen, 2012; Piserchia et al., 2017). Previous studies have also shown that monkeys trained to initiate a reaching movement by different sensory triggers (e.g., auditory tone, visual cue) recruit the same M1 neuron population (Lamarre et al., 1983). The prediction is that a common group of neurons in M1 should respond to mechanical and visual disturbances of the limb as well as visual disturbances of the goal.

Here, we explored these two hypotheses by training monkeys to make goal-directed reaches while disturbances to the limb and goal were applied. We demonstrate that proprioceptive feedback of the limb and visual feedback of the limb and the goal influence an overlapping group of neurons in M1 and evoke similar patterns of activity. However, there was a notable subpopulation of neurons unique to each perturbation that is inconsistent with either hypothesis suggesting a partial convergence of sensory modalities.

## Materials and Methods

The study involved two monkeys (*Macaque mulatta*, males, 17–20 kg) and was approved by the University's Research Ethics Board and Animal Care Committee. Monkeys were trained to place their upper limb in an exoskeleton robot (Kinarm).

### Lateral reaching task

**Figure 1.**
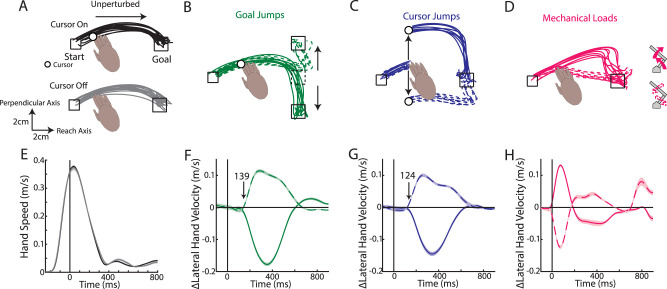
Example kinematics. ***A***, Example hand paths of Monkey M reaching for cursor-on (top) and cursor-off trials (bottom). ***B–D***, Example hand paths for goal jumps (***B***), cursor jumps (***C***), and mechanical loads (***D***). Solid and dashed lines are perturbations requiring corrections toward and away from the body, respectively. ***E***, The average hand speed on cursor-on and cursor-off trials. ***F–H***, The change in the lateral hand velocity for goal jumps (***F***), cursor jumps (***G***), and mechanical loads (***H***). Note, for the mechanical loads the change in lateral hand velocity starts at 0 ms due to the displacement caused by the loads. Arrows denote the onset of the kinematic correction using a difference function between the perturbations in opposite directions.

Monkeys were trained to make goal-directed reaches while countering unexpected perturbations to the limb or goal. At the beginning of a trial, the monkey placed and held their hand inside a start target (red square; length and width, 1.2 cm) for 750–1,500 ms. Then, a goal target (white square; length and width, 1.6 cm; joint configuration in middle of reach: shoulder, 30°; elbow, 87°) appeared lateral to the starting position that indicated the spatial location of the goal and provided the cue to initiate the reach. The reach primarily involved a shoulder and elbow extension motion and for Monkeys M and A, the goal targets were placed 10 cm and 8 cm from the start target, respectively. Monkeys had 1,400 ms to reach the goal and maintain their hand inside the goal for 500 ms to receive water reward. We included trials where visual feedback of the hand (white circular cursor; diameter, 1.6 cm) was provided for the entire trial duration and trials where visual feedback of the hand was removed 2 cm into the reach and reappeared 200 ms later. On random trials, we applied one of three perturbation types, goal jumps, cursor jumps, or mechanical loads. Mechanical loads consisted of torques applied to the shoulder and elbow joints in two opposite directions, one that flexed the shoulder and extended the elbow and the other that extended the shoulder and flexed the elbow. Shoulder and elbow torques were equivalent in magnitude and were 0.28 and 0.24 Nm for Monkeys M and A, respectively. Visual feedback of the hand was also removed for 200 ms after the mechanical load was applied. Cursor jumps consisted of displacements to the cursor's position perpendicular to the axis connecting the start and goal targets (Fig. 1*A*, reach axis). Two cursor jump directions were included that displaced the cursor away from or toward the body and the size of the displacement was 4 cm and 3 cm for Monkeys M and A, respectively. Goal jumps were identical to cursor jumps except that the goal's position was displaced. Note, visual feedback of the hand was provided continuously for both the cursor and target jumps. All perturbations were applied 2 cm into the reach. In a block of trials, monkeys performed eight unperturbed reaches with visual feedback of the hand, four reaches with visual feedback of the hand temporally removed for 200 ms, and six perturbation trials (two directions × three perturbation types). Monkeys completed 10–25 blocks in a recording session.

### Anterior reaching task

For a subset of sessions, monkeys also completed reaches to a goal located directly in front of the shoulder (anterior reach). These reaches followed the same timing parameters as the lateral reaches denoted above. Goal and cursor jumps were still in the direction that was lateral to the reach axis, which now resulted in jumps that were lateral or medial to the body. Mechanical loads were the same magnitude; however, now they either flexed the shoulder and elbow joints or extended the shoulder and elbow joints. In a recording session, monkeys completed 10–15 blocks of the lateral reaches followed by 10–15 blocks of the anterior reaches or completed the anterior reaches first followed by the lateral reaches. The ordering of the blocks was counterbalanced across sessions.

### Estimating visual onsets

There is an approximately 20–40 ms latency in the visual display between when a ,nd is sent to jump the cursor or goal and when it appears on the screen. On a trial-by-trial basis, we estimated the visual latency by fixing two photodiodes to the screen. When the goal or cursor jumped, two white squares would also appear that were positioned on the screen coincident with the photodiode placements. Jump onsets were estimated as the average onset of the two photodiodes or the onset detected by a single photodiode when the other photodiode signal was poor. On trials where a cursor and goal jump did not occur, the white squares still appeared at the same point in the reach so that we could align the unperturbed trials.

### Neural recordings

In each monkey, floating microelectrode arrays (96-channel, Utah arrays) were surgically implanted into the arm region of primary motor cortex. Surgery was performed under aseptic conditions and the arm region was identified by visual landmarks. During surgery, we used a dura substitute (GORE PRECLUDE Dura Substitute, W.L. Gore and Associates) that was placed over the array and the dura was reattached (GOR-TEX Suture, W.L. Gore and Associates). Spike waveforms were sampled at 30 kHz by either a 128-channel neural signal processor (Blackrock Microsystems) or a Grapevine processor (Ripple Neuro). Neural recordings were collected over five separate recording sessions in Monkey M and three separate recording sessions in Monkey A.

### Muscle recordings

In Monkey M, we surgically implanted a 32-channel chronic EMG system (Link-32, Ripple Neuro). This system had eight leads (impedance 20 kOhms) that could be inserted into the muscle with each lead having four separate contacts for recording muscle activity. Each lead was connected to an internal processor that was surgically implanted under the skin and located near the midline of the back at the midthoracic level. We implanted the brachioradialis, brachialis, the lateral and long heads of the triceps, biceps (long head), pectoralis major, and anterior and posterior deltoids. During a recording session, an external transmitter was attached on the skin over the internal processor and maintained in position by a magnet in the processor. The internal processor received power from the transmitter and transmitted the EMG signals transcutaneously. The signal was transmitted to the Grapevine processor, bandpass filtered (15–375 Hz), and recorded at 2 kHz. EMG recordings were collected over three separate recording sessions in Monkey M.

### Data analysis

#### Kinematic analysis

Kinematic signals were low-pass filtered with a sixth-order, zero-phase lag Butterworth filter (cutoff frequency 10 Hz). The endpoint of the reach was defined as the first time point after the peak hand speed that was <10% of the peak hand speed. Movement time was defined as the time duration between when the monkey left the start target and first entered the goal target.

#### EMG recordings

Muscle activity was down sampled to 1 kHz. For a given lead, we computed the differential signals between the two most proximal contacts and the two most distal contacts resulting in two differential signals from each recorded muscle. The differential signals were rectified and smoothed with a Butterworth low-pass filter with zero-phase lag at a cutoff frequency of 50 Hz. Muscle activity was aligned to perturbation onset or the equivalent onset on unperturbed trials and trial averaged. For muscle activity related to mechanical perturbations, we subtracted the activity on unperturbed reaches without visual feedback from the activity on mechanical perturbation reaches. For activity related to the visual perturbations, we employed the same method except using activity on unperturbed reaches with visual feedback. The muscle's preferred perturbation direction was determined for each perturbation type by calculating the activity with the largest perturbation response within the first 300 ms of the perturbation onset. Activity was normalized by the mean activity in the first 300 ms after the perturbation onset for each muscle signal.

#### Preprocessing neural recordings

Spike timestamps were convolved with a kernel approximating a postsynaptic potential (1 ms rise time, 20 ms fall time; Thompson et al., 1996) to estimate the instantaneous activities. Activities were aligned to perturbation onset following the same procedure as for muscle activities.

#### ANOVA analysis

For each neuron/muscle, we applied a three-way ANOVA with time epoch (levels: baseline epoch, −100 to 0 ms; perturbation epoch, 0–300 ms), perturbation direction (two levels), and perturbation type (levels: mechanical loads, goal jumps, and cursor jumps) as factors. Neurons/muscles were classified as “perturbation responsive” if there was a significant main effect for time or any interaction effects with time (*p* < 0.05; Bonferroni’s correction factor = 4). Neurons/muscles classified as significant were then subjected to separate two-way ANOVAs for each perturbation type with time and direction as factors. Neurons/muscles were classified as responsive for a given perturbation type if a significant main effect or interaction effect was found (*p* < 0.05; Bonferroni’s correction factor = 2).

#### Response range

The response range for a neuron was calculated for each perturbation type separately by taking the activity related to the correction toward the body and subtracting the activity related to the correction away from the body. The resulting activity was then averaged over the perturbation epoch.

#### Total least-square (TLS) regression

TLS regression was used to find a linear relationship between the response ranges from two perturbation types (Fig. 7). Ordinary least square (OLS) regression has been used in previous studies (Crammond and Kalaska, 2000); however, this method assumes one set of response ranges is the independent variable (i.e., no sampling noise; denoted as *x*) and thus only tries to find a line that minimizes the error between the dependent variable (*y*) and the line $y_{i}^{\mathrm{line}}=\min_{y_{i}^{\mathrm{line}}}(\sum_{i}{\left(y_{i}-y_{i}^{\mathrm{line}}\right)}^{2})$. In contrast, TLS regression does not assume any variables are independent and finds a line of best fit that minimizes the total error between each data point and the line $\left(y_{i}^{\mathrm{line}},x_{i}^{\mathrm{line}})=\min_{y_{i}^{\mathrm{line}},x_{i}^{\mathrm{line}}}(\sum_{i}{\left(y_{i}-y_{i}^{\mathrm{line}}\right)}^{2}+{\left(x-x_{i}^{\mathrm{line}}\right)}^{2}\right)$. TLS was performed by first subtracting the means for each response range $\left(\bar{y}\;\;\bar{x}\right)$ followed by singular value decomposition to find the slope (*m*). The left singular vector with the largest singular value was retained and the slope of the line of best fit was given as the ratio between the coefficient for the data on the *y*-axis over the coefficient for the data on the *x*-axis. The equation of the line of best fit is then $y^{\mathrm{line}}=m\cdot x^{\mathrm{line}}+b$ where $b=m\cdot \bar{x}+\bar{y}$. The significance of the slope was determined by shuffling the perturbations labels and recalculating the slope. This was repeated 1,000 times, and the probability value was calculated as the number of shuffled samples with slope smaller than the actual slope.

#### Onsets

The onset of perturbation-related activity was estimated by calculating the mean and standard deviation of the perturbation-related activity during the baseline period (100 ms before perturbation onset). The onset was then defined as the first time point to exceed the baseline mean by three standard deviations (positive or negative) for 20 consecutive time points. This method was used to calculate the onset for individual neurons, the neural population activity, and the muscle population activity. For individual neurons, the onset was only calculated once per neuron in the perturbation direction that elicited the largest absolute response from the unperturbed trials during the perturbation epoch.

To determine if the distribution of onsets for unimodal neurons (e.g., target-only neurons) were significantly different than random, we binned all neurons that had a detectable onset for the given perturbation (i.e., unimodal and multimodal) into 25-ms-wide bins (Fig. 5) and each bin's value was divided by the total number of neurons with a detectable onsets yielding a proportion of neurons for each time bin. These proportions were then multiplied by the total number of neurons that responded to a single perturbation yielding the expected number of neurons under random sampling for each time bin. This procedure was repeated for each individual perturbation type. To gain statistical power, we collapsed across monkeys and conducted a *χ*^2^ test to determine if the distribution of onsets for unimodal neurons were significantly different than the random distribution we calculated.

#### Average population activity

An average population response was calculated to estimate the total change in the network in response to the perturbations. We determined each neuron's preferred corrective movement by averaging its activity over the perturbation epoch. The corrective movement with the absolute largest change in activity from the unperturbed activity was then defined as the preferred corrective movement. If the change in activity was negative for a neuron in its preferred corrective movement, we multiplied its time series by negative one. This reduced the cancelling out of activity when averaging across the population of neurons.

#### Principal components analysis

Principal components analysis (PCA) was used to identify the low-dimensional subspace for the perturbation-related activity. For each perturbation type, we averaged each neuron's perturbation-related activity in nonoverlapping 10 ms windows to yield 30 time points for each perturbation direction. The activity of each neuron was soft normalized by its range (+5 sp/s) by finding its maximum and minimum activities during the perturbation epoch over all perturbation types (mechanical loads, goal jumps, cursor jumps). Note, the same normalization constant was applied to each perturbation type. We then constructed separate matrices for each perturbation type that were of size *N* × *DT*, where *N* is the number of neurons, *D* is the number of perturbation directions (2), and *T* is the number of time points (30). The mean activities in each row were then subtracted. Singular value decomposition was used to identify the principal components of the matrix, and the top 10 principal components were kept.

We used a cross-validated approach to draw a more accurate comparison between the amount of variance captured between perturbation types. For a given perturbation type, we randomly assigned trials into equally sized groups, and the same processing steps were applied as above. One group was used to calculate the principal components (trained) while the left-out group was used to calculate the amount of variance captured by those principal components. These principal components were also used to calculate the amount of variance accounted for by the other two perturbation types after randomly down-sampling trials to match the left-out group. This procedure was repeated 1,000 times for each perturbation type.

#### Overlap index

We quantified the overlap between the subspaces by calculating the overlap index from Rouse and Schieber (2018):

$$\mathrm{overlap}=\frac{\mathrm{tr}(\Sigma_{1}\Sigma_{2})}{||\Sigma_{1}|{|}_{F}||\Sigma_{2}|{|}_{F}},\;$$

where $\Sigma_{1}$and $\Sigma_{2}$ are the covariance matrices for perturbation types 1 and 2, tr is the trace operator, and $||\cdot |{|}_{F}\;$is the Frobenius norm operator. Activity was preprocessed the same way as for the PCA. The overlap index was computed between each pair of perturbation types.

The overlap index can range from 0, indicating no overlap between subspaces, and 1 indicating perfect overlap between subspaces. Confidence intervals were generated by randomly selecting half of the trials for each perturbation condition and calculating the subsequent overlap. This was repeated 1,000 times for each comparison between perturbation types.

We generated two null distributions for comparison. One distribution estimated the overlap between two independent samples from the same perturbation type (within-perturbation distribution). For a perturbation type, we split trials into two, equally sized groups and then calculated the overlap between these two groups following the same procedure as above. This was repeated 1,000 times for each perturbation type and overlap values were pooled. The second distribution compared how overlapping two samples were when the neuron labels were shuffled. For a perturbation type, we again split trials into two, equally sized groups. The neuron labels were then randomly shuffled in one group, and the overlap was then calculated between the two groups. This was repeated 1,000 times for each perturbation type and overlap values were pooled.

## Results

### Behavior, neural, and muscle activities are similar with and without visual feedback of hand position

**Figure 2.**
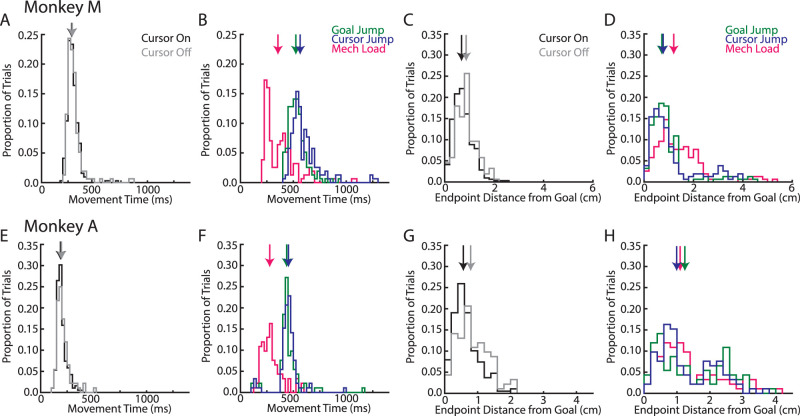
Movement times and endpoint distance from goal across monkeys. ***A***, Movement times for Monkey M for cursor-on and cursor-off unperturbed reaches. Movement time was defined as the time between when the hand left the start target and when the hand first contacted the goal target. Trials have been pooled across all recording sessions. Arrows denote the median of the distributions. Distributions for cursor-on and cursor-off trials were not significantly different (two-sample *t* test: *t*_(471)_ = 1.6; *p* = 0.12). ***B***, Same as ***A*** for perturbation trials. ***C***, Same as ***A*** except for the distance the reach endpoint was from the goal. Distributions for cursor-on and cursor-off trials were significantly different (*t*_(471)_ = 3.6; *p* < 0.001). ***D***, Same as ***C*** for perturbation trials. ***E–H***, Same as ***A–D*** for Monkey A. ***E***, Distributions for cursor-on and cursor-off trials were not significantly different (*t*_(279)_ = 1.9; *p* = 0.06). ***G***, Distributions for cursor-on and cursor-off trials were significantly different (*t*_(279)_ = 4.0; *p* < 0.001). Note, Monkey M had longer movement times than Monkey A due in part to Monkey M completing a 10 cm reach and Monkey A completing an 8 cm reach.

**Figure 3.**
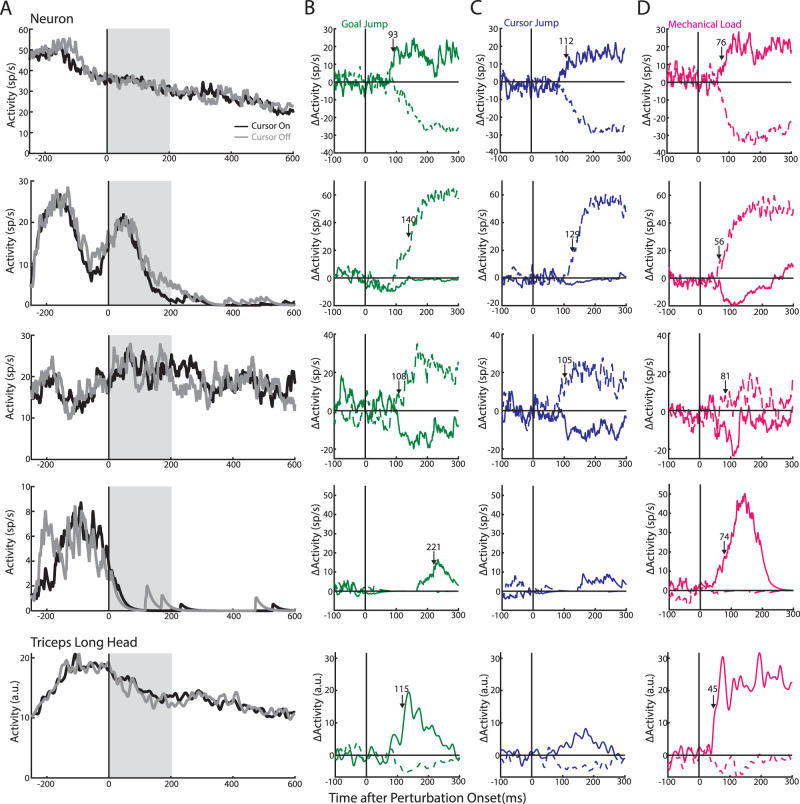
Example neuron activities. ***A***, Activities from four example neurons (first four rows) and muscle activity (bottom row) during reaches for cursor-on (black) and cursor-off trials (gray). Gray area demarcates when vision was removed. ***B–D***, The change in activities (ΔActivity) for the same four example neurons and muscle in response to the goal jumps (***B***), cursor jumps (***C***), and mechanical loads (***D***). Solid and dashed lines are responses to perturbations requiring corrections toward and away from the body, respectively. Arrows denote when a significant change in activity was detected using the difference function between perturbations in the opposite direction.

**Figure 4.**
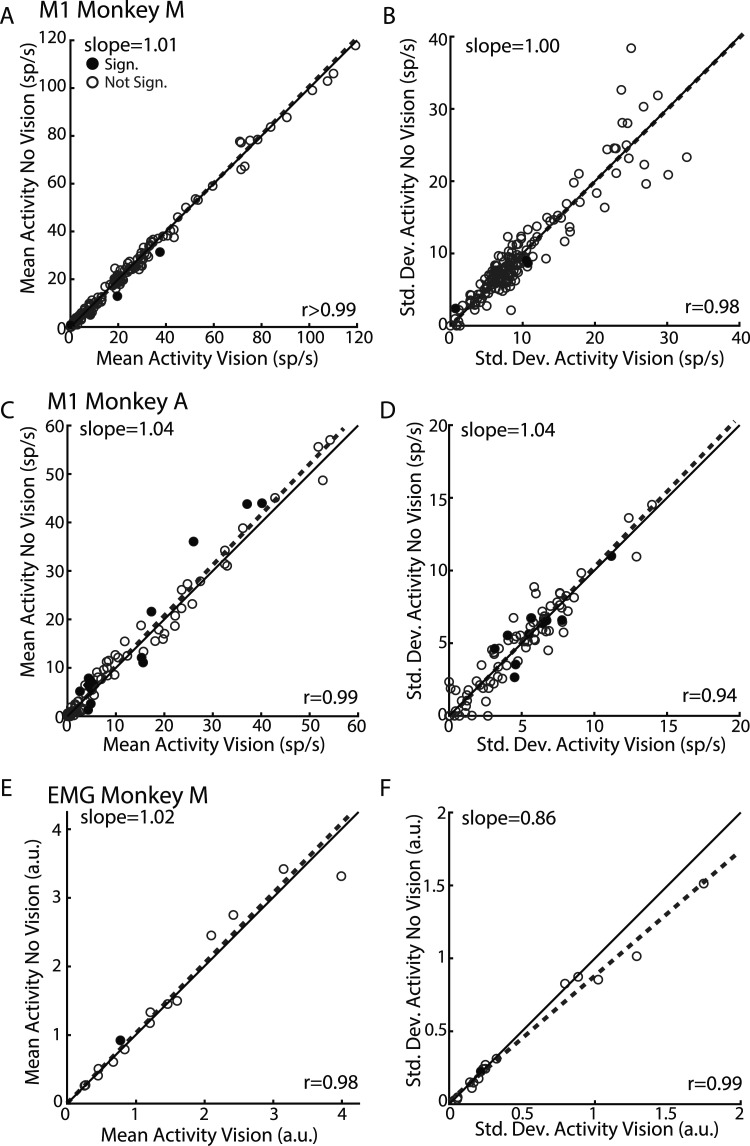
M1 activity is largely unaffected by removing cursor feedback. ***A***, For Monkey M, comparison of the mean activities during unperturbed reaches for cursor-on (abscissa) and cursor-off (ordinate) trials. Activity was averaged from 100 to 250 ms after the cursor feedback was removed. Each circle denotes one neuron. Dashed line reflects the line of best fit identified using total least squares regression (slope indicated in top left corner). ***B***, Same as ***A*** except for the standard deviation across trials. ***C***, ***D***, Same as ***A*** and ***B*** except for Monkey A. ***E***, ***F***, Same as ***A*** and ***B*** except for EMG from Monkey M.

We trained monkeys to reach to a goal and on random trials applied perturbations to either the goal or limb during the movement (Fig. 1*A–D*). For two perturbations, they involved either a jump to the visual feedback of the goal or visual feedback of the limb (Fig. 1*B*,*C*, white cursor). We also probed responses to proprioceptive feedback of the limb by applying a mechanical load that physically displaced the limb (Fig. 1*D*). To isolate the proprioceptive feedback response only, we transiently removed visual feedback of the hand (white cursor, removed for 200 ms) at the time of the mechanical load. In order to verify this transient removal of vision had minimal impact on performance, we compared unperturbed trials where cursor feedback was provided for the entire trial (cursor-on trials) with trials where cursor feedback was transiently removed (200 ms, cursor-off trials; Fig. 1*B*). We found cursor-on and cursor-off trials had similar movement times (Fig. 2*A*,*E*, time between leaving the start position and first contacting the goal target). However, there was an increase in endpoint distance from the goal for cursor-off trials (Monkey M|A, 0.87|0.79 cm) than that for cursor-on trials (0.68|0.57 cm) by ∼0.2 cm which likely reflects that our monkeys are highly experienced in this task (distance of the reach endpoint was from the goal; Fig. 2*C*,*G*). Further, we compared the magnitude of M1 activity (Monkey M|A, *n* = 172|85) between cursor-off and cursor-on trials. Figure 4, *A* and *C*, compares the mean M1 activity for cursor-off trials in the 200 ms when the cursor was removed (Fig. 3*A*, gray region) with activity for cursor-on trials with the equivalent time epoch. Neural activity was strongly correlated across neurons for both monkeys (*r* > 0.9) and regression slopes were near unity. Similarly, we examined the trial-to-trial variability between cursor-off and cursor-on trials for each neuron's activity in the same epoch and again found the variability was highly correlated (*r* > 0.9) with slopes near unity (Fig. 4*B*,*D*). Lastly, we examined muscle activity for cursor-off and cursor-on trials and found the mean and variability of muscle activity were strongly correlated (Fig. 4*E*,*F*) and with regression slopes near unity. Thus, transient removal of visual feedback of the limb had some impact on motor performance with decreased endpoint accuracy, while substantial differences in M1 and muscle activity were not detected.

### Monkeys rapidly counteract perturbations to the limb and goal

Next, we examined corrections for the different perturbation types (goal jumps, cursor jumps, and mechanical loads). Each perturbation type required corrections that moved the limb either toward the body (Fig. 1*B–D*, solid lines) or away from the body (dashed lines). Monkeys were able to quickly initiate a correction to each perturbation type within <200 ms of the perturbation (Fig. 1*F–H*). Perturbations resulted in longer movement times (24–138% increase; Fig. 2*B*,*F*) and greater endpoint distance (13–119% increase; Fig. 2*D,H*) than the unperturbed reaches.

Many neurons displayed robust responses following mechanical and visual perturbations with four example neurons shown in Figure 3*B–D*. The first neuron (Fig. 3*B*, top row) displayed a reciprocal response for goal jumps within 93 ms of the jump onset with an increase (solid) and decrease (dashed) in activities for corrective movements toward and away from the body, respectively. These changes in activity plateaued within 150 ms of the jump onset and remained relatively constant over the next 150 ms. However, the plateau for the inhibition response may reflect that the activity of the neuron was approaching 0 sp/s (Fig. 3*A*, top row). This neuron displayed a similar pattern of responses for cursor jumps (Fig. 3*C*, top row) and mechanical loads (Fig. 3*D*, top row). Neuron 2 (second row) displayed similar excitations for corrections away from the body across the different perturbation types. Neuron 3 (third row) displayed a similar pattern of responses across the two visual perturbations with an increase and decrease in activities for the corrective movements away from and toward the body, respectively. This neuron had similar selectivity for the mechanical loads; however, its responses were noticeably smaller. In contrast, neuron 4 (fourth row) exhibited considerably larger activity for the mechanical loads than either cursor jump or goal jump while still maintaining the same selectivity across perturbation types.

### Each perturbation type targets similar neurons in M1

**Figure 5.**
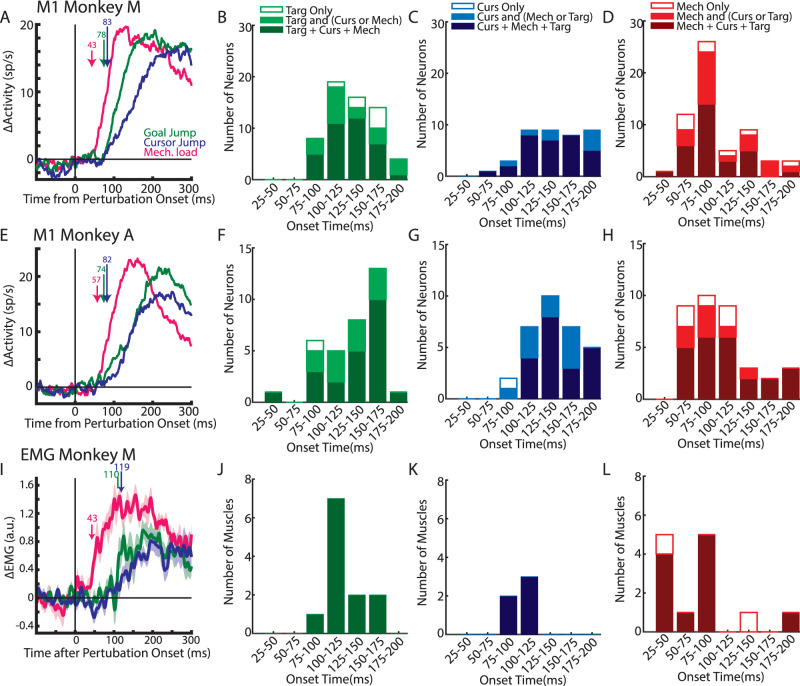
Proprioceptive feedback alters M1 activity earlier than visual feedback. ***A***, The average activity across neurons for Monkey M. Arrows indicate when a significant increase from baseline was detected. Only neurons with significant activity for at least one perturbation type were included. ***B***, The onset across individual neurons for the target jumps. Different colors of bars denote subsets of neurons. Dark green bars indicate neurons that responded to all three perturbation types (Fig. 6), light green bars denote neurons that responded to the target jump and only one other perturbation type (cursor jump or mechanical loads), and open bars denote neurons that responded to target jumps only. ***C***, ***D***, Similar to ***B*** for cursor jump and mechanical load onsets. ***E–H***, Same as ***A–D*** for Monkey A. ***I–L***, Same as ***A–D*** for muscle activity from Monkey M.

Our objective is to identify whether each feedback source recruited similar or independent groups of neurons in M1. We subtracted the unperturbed reach-related activity from each neuron's activity pattern for the different perturbation types leaving only the perturbation-related activity. This procedure was performed for perturbations in each direction. Mean activity was calculated for the baseline period prior to the perturbation (100 ms before perturbation onset) and during the perturbation (0–300 ms after perturbation onset) for all three perturbation types and perturbation directions. Significance testing was then performed on each neuron using a three-way ANOVA that included time (baseline and perturbation), perturbation type (three levels: mechanical, cursor, goal), and perturbation direction (two levels: toward and away from the body) as factors. For Monkeys M|A, we found 71|76% (*n* = 122|65) of neurons had a significant main effect for time or interaction effect(s) with time (*p* < 0.0125), which we labeled as perturbation-responsive neurons. We identified neurons that were responsive to a particular perturbation type by using a two-way ANOVA with time and perturbation direction as factors. Similar percentages of neurons were responsive for goal jumps (55|54%; *n* = 94|51), cursor jumps (44|60%; *n* = 75|51), and mechanical loads (55|60%; *n* = 94|46). These neurons received sensory feedback rapidly as the onset of perturbation-related activity at the population level occurred within <100 ms with responses to the mechanical loads arising earlier (Monkey M|A, 43|57 ms) than for either visual jump (goal, 78|74 ms; cursor, 83|82 ms; Fig. 5*A*,*C*). Similar results were found when examining individual onsets (Fig. 5*B–D*,*F–H*).

**Figure 6.**
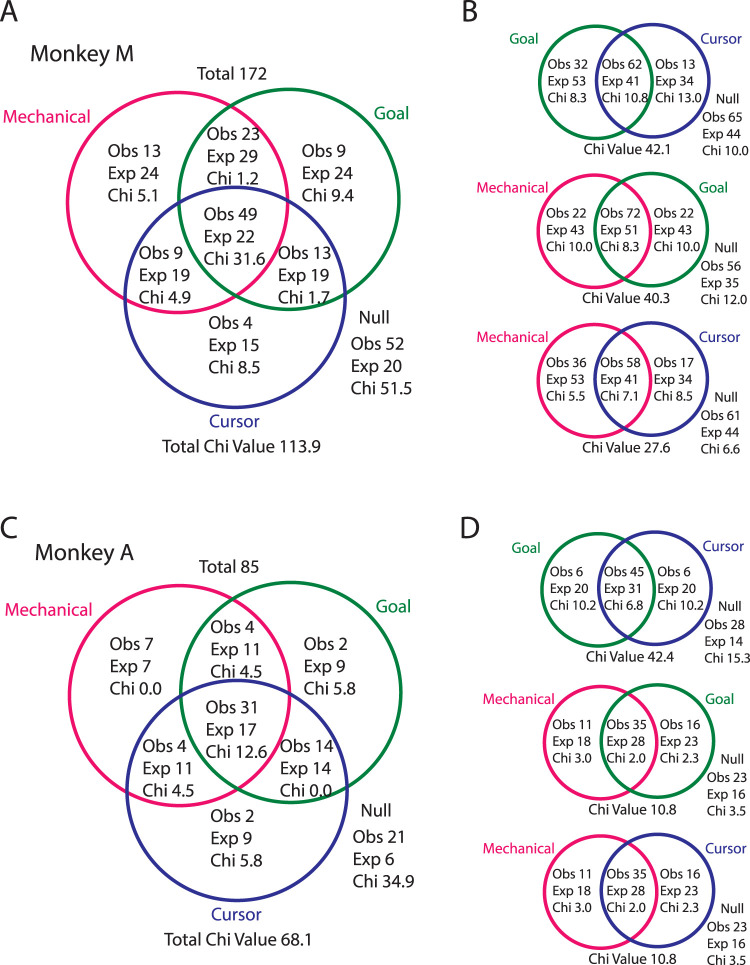
Each perturbation type influences overlapping neurons. ***A***, Venn diagram showing the number of neurons observed (Obs) in each class for Monkey M. The diagram also shows the number of expected (Exp) neurons assuming an independent distribution. Chi reflects the classes contribution to the total *χ*^2^ value ([Obs-Exp]^2^/Exp). ***B***, Venn diagrams classifying neurons using only two perturbation types for Monkey M. ***C***, ***D***, Same as ***A*** and ***B*** except for Monkey A.

From the percentages of neurons that responded to each perturbation type, we estimated the number of neurons expected to respond to zero, one, two, and three perturbation types assuming responses were independently assigned (expected distribution). Perturbation responses were significantly more overlapped than the expected distribution (Monkeys M|A, *χ*^2^ = 113.9|68.1; df = 4; *p* < 0.001|<0.001). In Monkey M|A, 15|13% (*n* = 26|11) of neurons responded to only one perturbation type, which was 2.4|2.4 times smaller than the expected distribution (Fig. 6*A*,*C*). In contrast, 28|36% (49|31) of neurons responded to all three perturbation types (common neurons), which was 2.6|3.4 times greater than the expected distribution.

Thus, there was substantial overlap between groups of neurons responsive to each feedback source. However, this finding may reflect a strong overlap between just two of the perturbation types or it could reflect an overlap among all three perturbation types. We repeated the analysis across pairs of perturbation types (Fig. 6*B*,*D*). Consistently, the number of neurons that responded to both perturbation types was 1.3–1.5 times greater than the expected distribution. In contrast, the number of neurons that responded to only one perturbation type was 1.5–3.4 times smaller than the expected distribution. Significant differences between the observed and expected distribution of neurons were found across all perturbation pairs (*χ*^2^ test; *p* < 0.01). Collectively, these results indicate that each perturbation type influenced an overlapping set of neurons in M1.

Next, we examined whether the different categories of neurons had distinct onset times. In particular, whether the neurons that responded to only one perturbation type (i.e., unimodal) responded earlier than neurons that responded to all three perturbations as this would provide support for the independent-input hypothesis. However, as shown in Figure 5*B–D*,*F–G*, neurons that responded to only one perturbation type (open boxes) had detectable onsets that were scattered throughout the 200 ms after the perturbation onset. Most units that responded the earliest to the perturbations were neurons that responded to two or three perturbation types (filled boxes). Extended Data Table 1 shows the number of unimodal neurons in 25 ms bins as well as the expected number of neurons assuming the onsets were assigned randomly (see Materials and Methods). Note, to increase statistical power, we collapsed across Monkeys M and A. *χ*^2^ tests revealed that neither the target-only (*χ*^2 ^= 2.4; df = 7) or mechanical-only (*χ*^2 ^= 4.1; df = 7) neurons had onsets that significantly differed from random. A *χ*^2^ tests for the cursor-only neurons was not applicable as only one neuron had a detectable onset. Thus, unimodal neurons did not have preferentially earlier onsets.

10.1523/ENEURO.0083-23.2024.t1 Table 1. Onsets for M1 neurons identified as responsive to target-only, cursor-only and mechanical-only perturbations. Neurons were pooled across both monkeys. Obs: observed, Exp: expected. Download Table 1, DOCX file.

### Neurons maintain their response ranges across perturbation types

A different way that each feedback source could independently influence M1 is by driving distinct activity patterns in the same neuron population. For example, a neuron may be strongly driven by one perturbation type but only weakly driven by a different perturbation type. At the extreme, neurons may even change their selectivity (i.e., tuning) for the perturbations: increase activity for the correction toward the body for one perturbation type but decrease activity for the same correction for a different perturbation type.

**Figure 7.**
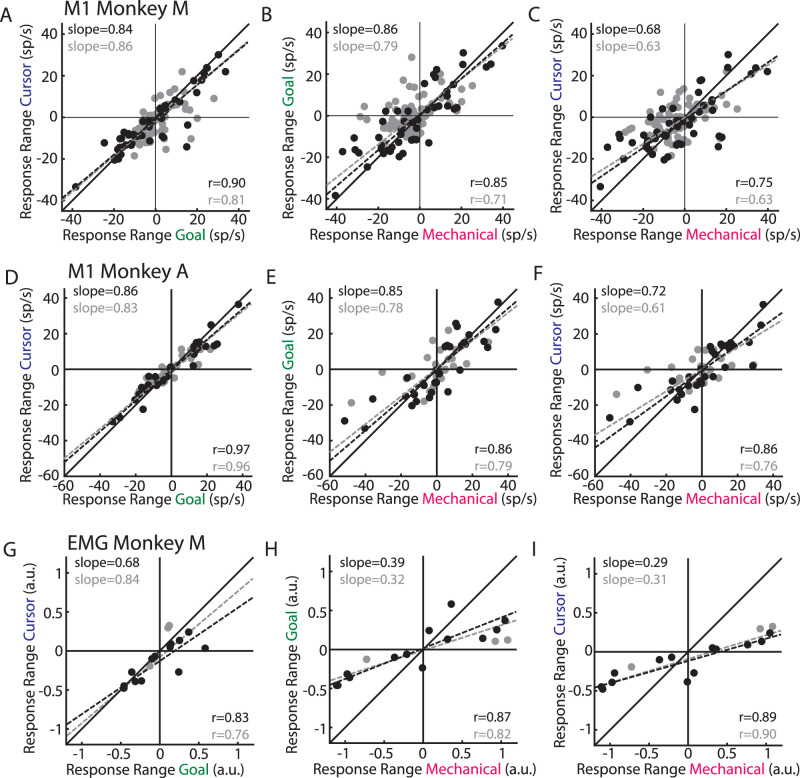
M1 neurons have similar response ranges across perturbation types. ***A***, Comparison of the response ranges between activities for the goal and cursor jumps. Black circles: neurons responsive to all three perturbation types. Gray circles: neurons responsive to at least one perturbation type. “*r*” is Pearson's correlation coefficient. Dashed lines reflect the line of best fit identified using total least squares regression (slope indicated in quadrant 2). ***B***, Same as ***A*** except comparing mechanical loads and goal jumps. ***C***, Same as ***A*** except comparing mechanical loads and cursor jumps. ***D–F***, Same as ***A–C*** except for Monkey A. ***G–I***, Same as ***A–C*** except for muscle activity from Monkey M.

Typically, examining the tuning of an M1 neuron involves characterizing multiple directions and fitting the activity to a cosine function. However, due to trial limits our perturbations only included two directions for each perturbation type. Thus, we characterized the response range by taking the difference between activities for the perturbations that required a correction toward the body from perturbations that required a correction away from the body (Fig. 3*B*, dashed subtracted from solid) and averaging the difference over the perturbation epoch (0–300 ms postperturbation). Note that this epoch likely reflects a combination of both sensory and motor processing. Neurons with greater responses for the corrections away from or toward the body will have positive or negative response ranges, respectively. Figure 7, *A* and *D*, compares the response ranges for goal- (abscissa) and cursor-related (ordinate) activities. Neurons responsive to all three perturbation types (black circles) resided near the unity line (solid line) and were highly correlated across the population (Monkey M|A, correlation coefficient *r* = 0.90|0.97; *p* < 0.001 for both). Note, neurons with response ranges lying along the unity line indicate neurons that reflect the motor correction rather than the sensory input which would lie along the line perpendicular to the unity line. The axes that captured the largest amount of variance (dashed black lines, total least squares regression) had a slope slightly less than unity (0.84|0.86) indicating that the responses for the cursor jumps were ∼15% smaller than the goal jumps (shuffle control *p* = 0.002|*p* < 0.001). We found significant but noticeably weaker correlations when comparing the response ranges between the mechanical-related activities (abscissa) and activities related to either visual perturbation (ordinate; Fig. 7*B*,*C*,*E*,*F*; mechanical with goal *r* = 0.85|0.86; mechanical with cursor *r* = 0.75|0.86; *p* < 0.001 for all). The slope was less than unity (mechanical with goal slope = 0.86|0.85; mechanical with cursor slope = 0.68|0.72) indicating that the responses for the visual perturbations were ∼22% smaller than those for the mechanical loads. Inclusion of all perturbation-responsive neurons yielded similar results (Fig. 7, gray circles).

From the response ranges, we could determine if neurons maintained their selectivity (i.e., preferred direction) for corrective movements across perturbation types. These neurons resided in the first and third quadrants of Figure 7, and we found a large majority of neurons maintained their selectivity across all three perturbation types (neurons responsive to all three perturbation types, Monkey M|A 82|87%; all perturbation-responsive neurons, 70|72%). Collectively, these results indicate that each feedback source had similar influences on individual M1 responses.

We repeated our analysis of the response range while examining a shorter time window near the onset of the earliest evoked M1 activity. For visual perturbations, we examined the response range in the 75–125 ms epoch, and for the mechanical perturbations, we examined the response range in the 50–100 ms epoch (Extended Data Fig. 1). Similar to the previous analysis, each pairwise comparison revealed response ranges that were positively correlated. For the comparison of the goal- and cursor-related activity, the axis that captured the largest amount of variance also had a positive slope; however, these slopes were noticeably less than unity which likely reflects the weaker onset of the cursor-related activity (Fig. 5). Similarly, when comparing the mechanical-related activity to either visual-related activity, we found the axis that captured the largest amount of variance had positive slopes albeit reduced indicating stronger mechanical-related activity than either visual-related activity. Collectively, these results indicate similar trends in the response range were also found for the earliest evoked perturbation responses.

10.1523/ENEURO.0083-23.2024.f1 Figure 1. **M1 neurons have similar response ranges across perturbation types during the onset of evoked activity.** Data are presented the same as Figure 7 except response ranges were calculated for the visual perturbations from 75-125ms after perturbation onset and for the mechanical perturbations from 50-100ms after the perturbation onsets. Download Figure 1, TIF file.

**Figure 8.**
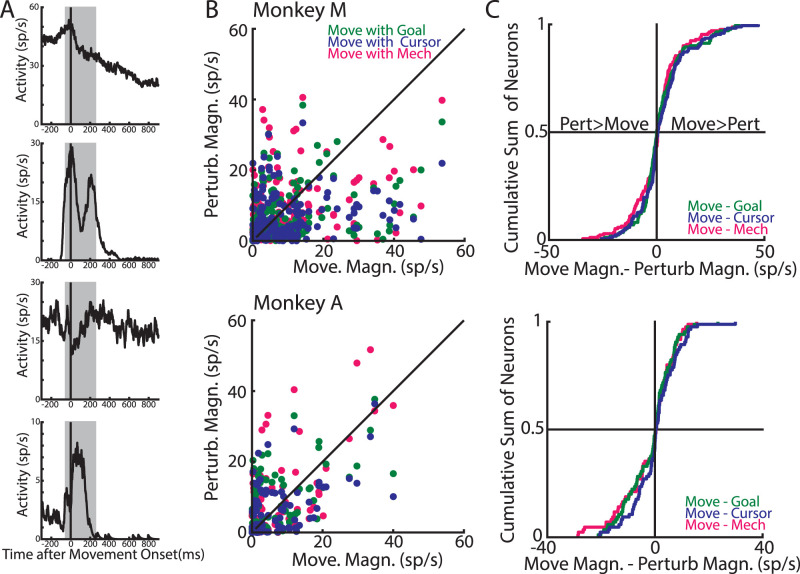
Perturbation-related activity is comparable to activity during baseline reaching. ***A***, Activities of the same four example neurons in Figure 2 during unperturbed reaches aligned to movement onset (5% max hand speed). Shaded area denotes the movement epoch (−50 to 250 ms). ***B***, Scatter plot comparing the absolute magnitude of movement-related activity with the magnitude of the perturbation-related activity. ***C***, Cumulative sums of the difference in the magnitudes of the movement-related and perturbation-related activities across cells.

Next, we wanted to contextualize the relative size of the response range by comparing it to the movement-related activity during unperturbed reaching (Figs. 3, 8*A*). Figure 8*B* compares the magnitude of the movement-related activity during unperturbed reaching (aligned to movement onset, movement epoch −50 to 250 ms after movement onset) with the magnitude of the response range for perturbed reaches. We found approximately equal number of neurons had either larger perturbation-related activities or movement-related activities (Fig. 8*B*,*C*). Thus, the perturbation-related activity was comparable in magnitude to the activity required to generate the initial reaching movement.

### Overlap between mechanical- and visual-related M1 activity patterns at the population level

Our results so far demonstrate that each feedback source targets a largely overlapping population of M1 neurons and that individual neuron responses are generally similar across feedback sources. However, recent studies have demonstrated that the same neuron population can represent different types of information independently by sequestering the information into orthogonal subspaces (Kobak et al., 2016; Ames and Churchland, 2019; Heming et al., 2019; Keemink and Machens, 2019; Cross et al., 2020). For example, neurons in M1 have similar tuning for reach direction during preparation and execution (Crammond and Kalaska, 2000). However, these activity patterns reside in orthogonal subspaces (Kaufman et al., 2014; Elsayed et al., 2016). Thus, for the independent-input hypothesis, each perturbation type may evoke an activity pattern that resides in an orthogonal subspace with respect to the other two perturbation types.

We explored this hypothesis by using principal component analysis (PCA) to identify the low-dimensional subspace each perturbation-related activity resided in. We found the top 10 principal components trained on the goal-related activity captured 55|73% of the variance for Monkeys M|A, respectively (Fig. 8*A*,*E*). If goal-related activity patterns differed substantially from the activity patterns evoked by the cursor and mechanical perturbations, then we would expect these principal components to capture very little variance of the cursor and mechanical evoked activity. However, we found the opposite as these components captured 44|65% of the variance for the cursor-related activity for Monkeys M|A. Likewise, the components captured 36|43% of the variance for the mechanical-related activity for Monkeys M|A. Repeating this analysis using components identified from the cursor-related activity revealed that these components captured 49|69% of the variance for the cursor-related activity while also capturing 36|43% of the variance for the goal-related activity and 30|45% of the variance for the mechanical-related activity for Monkey M|A (Fig. 8*B*,*F*). Lastly components identified from the mechanical-related activity revealed that these components captured 59|74% of the variance for the mechanical-related activity while also capturing 35|40% of the variance for the goal-related activity and 32|40% of the variance for the mechanical-related activity for Monkeys M|A (Fig. 8*C*,*G*).

**Figure 9.**
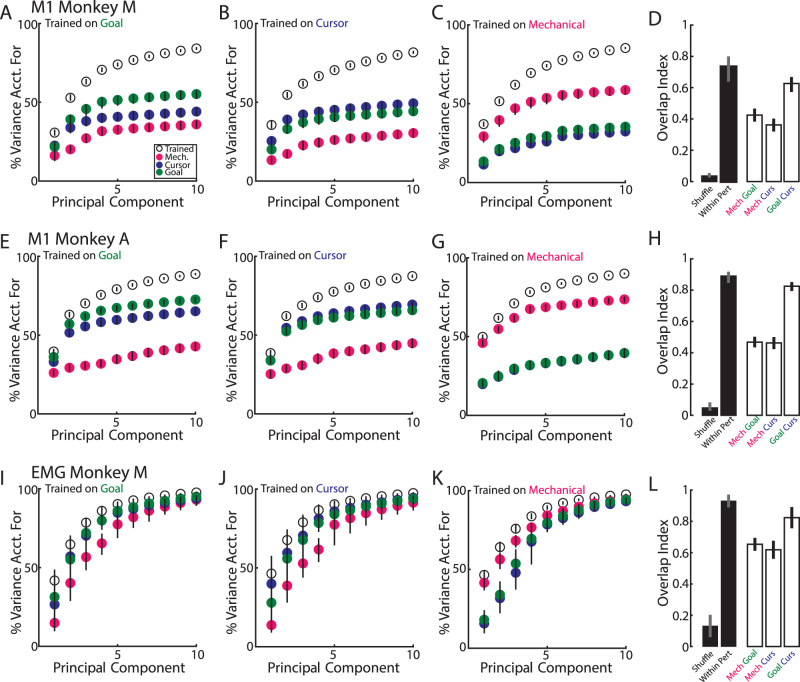
Activity patterns overlap across perturbation types. ***A***, Variance accounted for by the top goal–jump principal components for Monkey M. Data are presented as a cumulative sum showing how inclusion of each principal component increases the total variance captured for each perturbation type. Circles and bars denote the median and the 5th and 95th percentiles of the distributions. ***B***, ***C***, Same as ***A*** for cursor jumps and mechanical loads. ***D***, Overlap index between perturbation types (clear bars) and the shuffle and within-perturbation distributions (filled bars). Bars denote the median and 5th and 95th percentiles of the distribution. ***E–H***, Same as ***A–D*** except for Monkey A. ***I–L***, Same as ***A–D*** except for EMG from Monkey M.

Another approach to quantify the similarity in the population structure between feedback sources is by calculating the overlap index (Rouse and Schieber, 2018). The overlap index ranges from 0, indicating no overlap between subspaces (i.e., orthogonal and separate subspaces), to 1 indicating perfect overlap (i.e., the same subspace). For comparison, we generated a null distribution that compared how overlapping two subspaces were after randomly shuffling neuron labels (shuffle). We also generated a null distribution that quantified the maximum overlap expected given sampling noise by calculating the overlap between two independent samples from the same perturbation type (within-perturbation distribution). The overlap between goal- and cursor-related activities was large (Monkeys M|A, 0.63|0.82; Fig. 9*D*,*H*) and was close to the within-perturbation distribution (0.73|0.89), though it was still significantly smaller (*p* = 0.03|0.01). The overlap between the mechanical-related and visual-related activities was smaller than the within-perturbation distribution (mechanical with goal, 0.42|0.47; mechanical with cursor, 0.36|0.46; within-perturbation *p* < 0.001 for all); however, they were still significantly greater than the shuffled distribution (*p* < 0.001). Collectively, these results indicate each perturbation type evoked similar population-level structure.

### Muscle activity exhibits similar overlap between perturbation types as M1 activity

Next, we examined the change in muscle activity in response to the different perturbation types. We identified the perturbation-related activity for each muscle by subtracting muscle activity from unperturbed trials from muscle activity from perturbation trials. We found a significant change in muscle activity between baseline (100 ms before perturbation) and the perturbation epoch [300 ms after perturbation epoch in 81% (*n* = 13), 88% (14), and 100% (16) of muscle samples for the goal jumps, cursor jumps, and mechanical loads, respectively (paired sample *t* test; Fig. 3*B–D*, bottom row)]. There was a strong correlation between response ranges during the perturbation epoch for the goal- and cursor-related activities (*r* = 0.83; *p* < 0.001; Fig. 7*G*) and the slope was less than unity (slope, 0.68) indicating responses for the cursor jump were 32% smaller than those for the goal jump. We also found strong correlations between the mechanical-related response ranges and the response ranges for either type of visual disturbance (Fig. 7*H*,*I*; mechanical with goal *r* = 0.87; mechanical with cursor *r* = 0.89; *p* < 0.001 for both). However, we found the slopes were considerably smaller than unity (mechanical with goal, 0.39; mechanical with cursor, 0.29) indicating that muscle activity for the visual perturbations were ∼66% smaller than that for the mechanical loads. As expected, almost all (except one) of the muscle recordings maintained their selectivity (i.e., preferred direction) across all perturbation types.

Next, we examined the population-level patterns spanned by the muscle activity. Figure 9*I* shows the top 10 goal principal components for muscle activity. Unlike neural activity, these 10 components captured nearly all of the variance for the goal jump, cursor jump, and mechanical loads. This is due to the smaller number of muscles recorded as the entire space of muscle patterns occupies a maximum of 16 dimensions. In contrast, neural activity can occupy 172 and 85 dimensions for Monkeys M and A, respectively. We mitigated this problem by restricting our observations to the top 3 components as three components captured a similar amount of variance from the training data (range, 82–84%) as the 10 components captured for the neural activity (82–90%). We found the top 3 goal principal components captured a substantial amount of the goal- (76%) and cursor-related (74%) muscle variance but captured slightly less of the mechanical-related variance (68%). Similarly, the top 3 cursor principal components captured a substantial amount of the cursor- (77% Fig. 9*J*) and goal-related (73%) muscle variance but captured less of the mechanical-related variance (61%). Lastly, the top 3 mechanical principal components captured a substantial amount of the mechanical-related muscle variance (84%; Fig. 9*K*) but captured less of the muscle variance for either visual perturbation (goal, 59%; cursor, 58%).

We computed the overlap index between muscle responses and found results that were similar to M1 activity (Fig. 9*L*). There was a high overlap between the goal- and cursor-related activities (0.82) that was comparable with the within-perturbation distribution (0.93), though still significantly smaller (*p* = 0.02). We also found a partial overlap between the mechanical-related activity and the visual-related activities (mechanical and goal, 0.65; mechanical and cursor, 0.62), which were significantly greater than the shuffle distribution (overlap, 0.13; *p* < 0.001). Collectively, these analyses indicate that different patterns of muscle activity were needed to correct for each perturbation type which could explain the partial overlap observed between the mechanical- and visual-related M1 activities.

### Overlap across perturbation types emerges rapidly with perturbation-related activity

**Figure 10.**
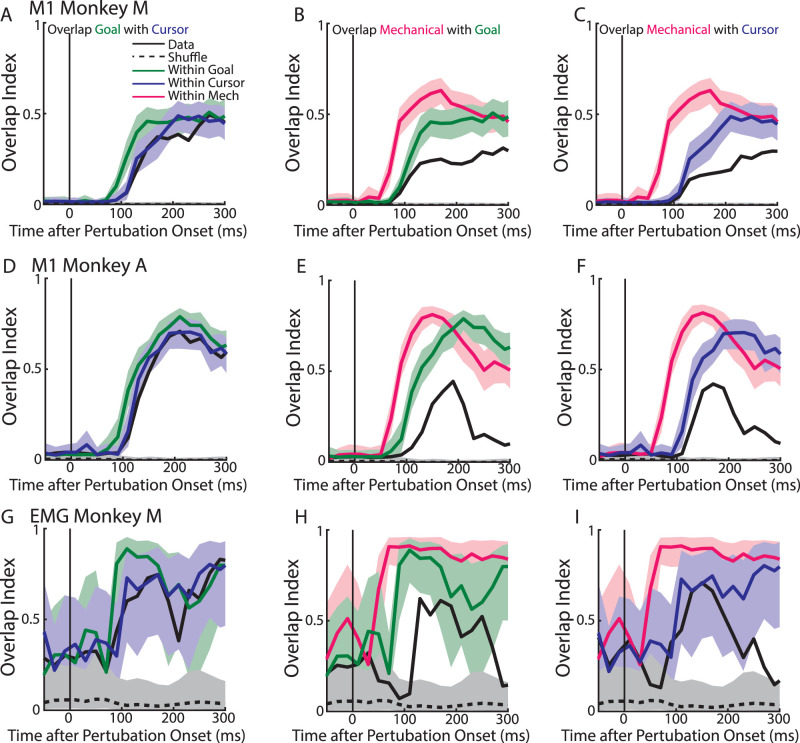
Time course of overlap index. ***A***, Time series of the overlap index between goal and cursor jumps (black solid line) for Monkey M. Activity was binned every 20 ms. The time series was also repeated for the shuffle distribution (black dashed line) and the within-perturbation distributions for the goal-related (green line) and cursor-related (blue line) activities. ***B***, Same as ***A*** except comparing mechanical loads with goal jumps. ***C***, Same as ***A*** except comparing mechanical loads with cursor jumps. ***D–F***, Same as ***A–C*** except for Monkey A. ***G–I***, Same as ***A–C*** for EMG signals. Prior to overlap calculation, EMG signals were filtered with a low-pass third-order Butterworth filter (cutoff 50 Hz). Note, the substantial overlap before perturbation onset is in part due to the small subspace spanned by EMG signals.

Next, we examined how the overlap evolved over time between the different perturbation types. One possibility is that each feedback source is initially represented independently by the motor system before being gradually integrated (Franklin et al., 2016; Oostwoud Wijdenes and Medendorp, 2017). Thus, the prediction is that the overlap between perturbation types should gradually emerge. We calculated the overlap index every 20 ms over the perturbation epoch (Fig. 10*A–F*). We found the overlap index between the goal- and cursor-related M1 activities emerged within ∼100 ms (Fig. 10*A*,*D*, black line) postperturbation and was comparable to the within-perturbation distributions of the goal-related (green line) and cursor-related activities (blue line). Further, the overlap between the mechanical- and visual-related M1 activities emerged within ∼100 ms of the perturbation onset (Fig. 10*B*,*C*,*E*,*F*). Note that the increase in the overlap index proceeded the within-perturbation onset for the mechanical loads (red line) reflecting that M1 responds earlier for mechanical loads than visual jumps (Fig. 5*A*,*C*). Interestingly, there was a small delay in the overlap between the mechanical and visual perturbations for Monkey A (Fig. 10*E*,*F*) which may reflect a small time window of integration. Similar trends were found in the muscle activity (Fig. 10*G–I*). Thus, the overlap between perturbation types emerged rapidly in the network.

### Overlap is still present when examining other movement directions

**Figure 11.**
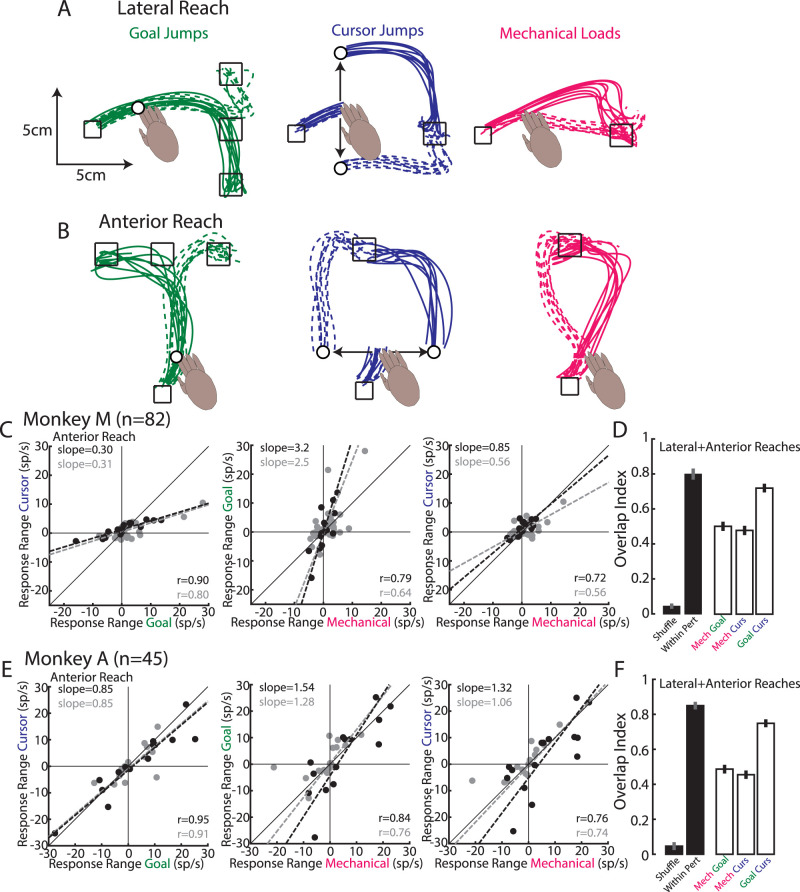
Overlap across perturbation types with increased perturbation directions. ***A***, Monkey M's lateral reaches following goal jumps (left), cursor jumps (middle), and mechanical loads (right). Same as Figure 1*B–D*. ***B***, Same as ***A*** except now for Monkey's M anterior reaches. ***C***, Response ranges comparing perturbation types for the anterior reaches. Data presented the same as in Figure 5. “*n*” denotes the number of recorded neurons. ***D***, Overlap index presented the same as Figure 6. ***E***, ***F***, Same as ***C*** and ***D*** for Monkey A.

One concern is whether we adequately characterized M1's responses to each perturbation type as we sampled from only two perturbation directions. This seems unlikely as previous work has shown that a greater proportion of M1 neurons respond maximally to perturbations that involve either combined shoulder flexion and elbow extension (whole-arm extension for corrections away from body) or combined shoulder extension and elbow flexion (whole-arm flexion for corrections toward the body; Cabel et al., 2001; Scott et al., 2001; Kurtzer et al., 2006; Lillicrap and Scott, 2013). Nonetheless, we verified that sampling from more perturbation directions yielded virtually the same overlap. Monkeys completed separate blocks of the same lateral reach (Fig. 11*A*) and also blocks of a sagittal reach starting from near the body and reaching to a distant goal (Fig. 11*B*). For the sagittal reach, the perturbations required a corrective movement that either flexed the shoulder and elbow joints (Fig. 11*B*, solid lines) or extended the shoulder and elbow joints (dashed lines). The perturbations for the lateral and sagittal reaches yielded four perturbation directions for each perturbation type. We found response ranges were correlated between perturbation types with the strongest correlation between goal jumps and cursor jumps (Fig. 11*C*,*E*, response range for sagittal reach shown only, Monkeys M|A, *n* = 82|45). For the sagittal reach, activity related to goal jumps tended to be larger than activity related to cursor jumps or mechanical loads. Critically, we found the overlap between goal- and cursor-related activities was substantial (Monkeys M|A, 0.72|0.75; Fig. 11*D*,*F*) and was close to the within-perturbation distribution (0.80|0.85), though it was still significantly smaller (*p* = 0.01|<0.001). The overlap between the mechanical-related activity with either visual-related activity was smaller than the within-perturbation distribution (mechanical with goal, 0.50|0.49; mechanical with cursor, 0.48|0.45; within-perturbation *p* < 0.001 for all). However, it was still significantly greater than the shuffled distribution (*p* < 0.001).

## Discussion

We identified how feedback responses from different sensory sources recruit M1 circuits during a goal-directed reaching task. Our results indicate sensory perturbations evoked a rapid and robust change in M1 activity which likely reflects both sensory processing and the corrective motor ,nds. Perturbations to visual feedback of the goal and hand and mechanical perturbations that probed proprioceptive feedback of the limb recruited similar pools of M1 neurons which had similar response magnitudes and directions. However, there was a stronger similarity of responses when comparing the two visual perturbations together than when comparing the mechanical perturbation with either visual perturbation. Collectively these results highlight how different sensory errors recruit partially overlapping circuits in M1. The overlapping portions of the circuit may reflect a response based on multisensory integration of feedback sources whereas the circuits distinct to each perturbation type may reflect a unisensory feedback response for each type of sensory error.

Perturbations to vision and proprioception had rapid and potent influences on M1 processing. A small majority of neurons responded to proprioceptive (58%) feedback consistent with previous studies (Rosén and Asanuma, 1972; Conrad et al., 1975; Lemon et al., 1976; Wong et al., 1978; Fetz et al., 1980; Lemon, 1981b; Fromm et al., 1984; Hummelsheim et al., 1988; Bauswein et al., 1991). A similar percentage of neurons responded to visual feedback of the limb (52%) and goal (55%). Visual and mechanical disturbances required corrections of 3–4 cm, and the corresponding activity in M1 was comparable in magnitude to the activity that initiated the 8–10 cm reach. Proprioceptive and visual feedback influenced M1 activity within ∼50 and ∼80 ms of a disturbance, respectively. The longer delay for vision is partly due to processing time of the retina as the lateral geniculate nucleus, an area immediately downstream of the retina, responds to visual input within ∼20–30 ms (Maunsell et al., 1999). In contrast, muscle spindles respond to a muscle stretch within ∼3 ms (Schäfer et al., 1999), and the conduction delay to first-order thalamic nuclei is approximately 6 ms (Lemon and van der Burg, 1979). Thus, sensory feedback has a potent influence on M1 when responding to external disturbances, and it is likely that sensory errors generated during natural reaching likely also have a potent influence (Crevecoeur et al., 2012; Crevecoeur and Kurtzer, 2018; Takei et al., 2018).

Interestingly, the timing for proprioceptive feedback was noticeably longer than previous studies that demonstrate M1 responds within ∼20 ms of a mechanical load during postural tasks (Evarts and Tanji, 1976; Wolpaw, 1980; Fromm et al., 1984; Boudreau and Smith, 2001; Pruszynski et al., 2014; Omrani et al., 2016). This may reflect differences in sensory processing between posture and reaching. Alternatively, this may reflect different subdivisions of M1 as the present study recorded neurons using a multielectrode array and thus sampled neurons only from the gyrus of M1 (rostral M1). In contrast, previous studies commonly recorded neurons from the gyrus and the caudal portion of M1 residing in the central sulcus. Previous work suggest that there are gradients along the rostral–caudal axis of M1 for anatomical and physiological features (Crammond and Kalaska, 1996, 2000; Cisek et al., 2003; Rathelot and Strick, 2009; Witham et al., 2016), and thus, faster timing may reside in neurons sampled from caudal M1.

We set out to identify how different sensory sources recruit M1 circuits. Our initial hypotheses considered two extreme possibilities, (1) different sensory errors recruit different circuits in M1 (independent hypothesis) or (2) different sensory errors recruit the same circuits in M1 (converge hypothesis). Our findings highlight how visual feedback of the limb and visual feedback of the goal evoked highly similar patterns of M1 activity more consistent with the convergence hypothesis. Examination of the population-level analysis (Figs. 9, 10) highlights how goal and cursor jumps evoked activity patterns in subspaces that were highly similar and differed only slightly more than comparing trials from the same perturbation type (i.e., the within-perturbation). At the single-neuron level, we found a large group of neurons that responded to both the cursor and goal jumps (Fig. 7) and exhibited similar activity patterns (Fig. 6).

However, it is worth noting that there was a substantive population of neurons that had significant responses to only one of the visual perturbations (Fig. 6*B*,*D*). It is interesting that despite these distinct populations of neurons, only a small difference was detected in the low-dimensional neural subspace. This may indicate that population-level analysis may miss important characteristics found at the single-neuron level. Alternatively, unique neuron populations may be an artifact in part caused by binary classification of neurons using an ANOVA approach.

A clearer divergence from the two extreme hypotheses is highlighted when contrasting perturbations of vision and proprioception. At the single-neuron level, proprioceptive and visual perturbations modulated overlapping populations of neurons with ∼43% of perturbation-sensitive neurons responding to all three perturbations which is considerably more than random chance (∼16%). This overlap was also observed at the population-level where each perturbation evoked activity that occupied overlapping neural subspaces. However, the overlap was considerably weaker than the cursor- and goal-jump comparison. It is possible that the weaker overlap may in part reflect differences in the required motor output as muscle activity evoked by visual and proprioceptive perturbations were not as strongly overlapped as the overlap between the cursor- and goal-jump muscle activity patterns (Fig. 9*I–L*). As such these results may reflect a lower bound on the convergence of visual and proprioceptive feedback responses in M1. Collectively, these results suggest a partial overlap between proprioceptive and visual responses, thus residing in the middle of the convergent input hypothesis and independent input hypothesis.

Our results contrast with previous studies examining cutaneous and proprioceptive feedback responses in M1 (Rosén and Asanuma, 1972; Lemon et al., 1976; Wong et al., 1978; Fetz et al., 1980; Tanji and Wise, 1981; Strick and Preston, 1982; Picard and Smith, 1992). These studies identify unique and largely independent circuits in M1 that are responsive for cutaneous and proprioceptive feedback with only a few neurons that were multimodal. Thus, our findings of strong overlap of rapid proprioceptive and visual feedback responses in M1 are not trivial and highlight targeting by each modality in M1.

One possible explanation for finding groups of neurons that were responsive to only one perturbation type is that they reflect an initial corrective response that only considers unimodal sensory input which is then followed by a corrective response based on multimodal input (Oostwoud Wijdenes and Medendorp, 2017). However, we believe this is not the case as unimodal responses emerged around the same time as the multisensory neurons suggesting parallel processing of unimodal and multimodal information. Note, these data do not refute this two-stage approach but suggest that it must rely on a different mechanism than distinct neuron populations. Instead, these responses may reflect that unimodal information may be required for computations required to support the ongoing reaching movement. For example, proprioceptive-only neurons may reflect a limb-only representation needed to map a limb-based difference vector to motor ,nds (Sober and Sabes, 2003, 2005).

The partial overlap across modalities could be a signal reflecting the integration of sensory feedback performed by areas upstream of M1 including frontal and parietal cortices. These areas receive proprioceptive and visual feedback with neurons that respond to both sensory modalities (Rizzolatti et al., 1981a,b; Snyder et al., 1998; Bakola et al., 2010; Omrani et al., 2016; Gamberini et al., 2017) and are likely involved with estimating a common limb state (Desmurget and Grafton, 2000; Shadmehr and Krakauer, 2008; Scott, 2012; Takei et al., 2021). Neurophysiological investigations also indicate that these areas are involved with generating a movement vector by combining limb and goal feedback (Snyder et al., 1998; Buneo et al., 2002; Pesaran et al., 2006; McGuire and Sabes, 2011; Bremner and Andersen, 2012; Piserchia et al., 2017).

Consistent with upstream state estimation is that M1 activity was largely unaffected by the removal of cursor feedback. Other groups also found that the motor system was insensitive to the removal of cursor feedback but interpreted this as evidence that reaching involves a ballistic phase where feedforward motor ,nds transport the limb toward the goal with little influence from sensory feedback (Woodworth, 1899; Meyer et al., 1988; Suway and Schwartz, 2019). However, our perturbations show M1 is still sensitive to sensory feedback inconsistent with this ballistic interpretation. The insensitivity to cursor visibility likely reflects that the motor system also uses internal and proprioceptive feedback to compensate for missing visual information consistent with multisensory state estimation (Crevecoeur et al., 2016; Kasuga et al., 2022). This compensation strategy is likely necessary as gaze shifts and blinks can disrupt the visibility of the hand during motor actions. Further, we found a small increase in the distance the reach endpoint was from the goal when cursor feedback was removed suggesting only a partial compensation by these alternative feedback sources.

There was also a noticeable difference in the relative magnitudes for visual and mechanical perturbations between M1 and muscles. Peak M1 activity was similar for mechanical loads and visual jumps of the cursor or target, whereas muscle activity was twice as large for mechanical loads than visual jumps (Fig. 5). This suggests M1 contributes ∼50% of the total motor output for loads with the remaining output likely generated by subcortical circuits including at the spinal level (Mewes and Cheney, 1991; Soteropoulos et al., 2012; Herter et al., 2015; Soteropoulos and Baker, 2020). However, this estimate has many assumptions. First, activity recorded in rostral M1 is representative of descending cortical control, in general. For example, it is unclear whether this relative sensitivity to proprioceptive and visual feedback will remain for caudal M1 where proprioceptive responses tend to be greater (Porter and Lemon, 1993). Second, neural responses for visual and mechanical disturbances contribute similarly to descending signals or output-potent spaces (Kaufman et al., 2014; Stavisky et al., 2017). This assumption seems reasonable as the population-level structure was similar between mechanical and visual perturbations. Finally, we likely underestimated the subcortical contribution to mechanical loads as the comparison between mechanical and visual perturbations assumed M1 was the only circuit involved with generating visual responses. Visual responses may involve subcortical circuits including the superior colliculus (Alstermark et al., 1987; Day and Brown, 2001; Pruszynski et al., 2010; Corneil and Munoz, 2014; Cross et al., 2019; Kozak et al., 2019). While comparing visual and mechanical responses at the muscle and neural levels provides a potentially important approach to probe cortical versus subcortical contributions to feedback corrections, further studies are required to address the inherent assumptions.

Feedback processing at cortical and subcortical levels highlight the hierarchical organization of the motor system with multiple feedback loops including transcortical feedback through M1 which is the highest level for online continuous control (Porter and Lemon, 1993; Schweighofer et al., 1998; Loeb et al., 1999; Todorov et al., 2005; Liu and Todorov, 2009; Merel et al., 2019). Current theories inspired by engineering principles have adopted a serial approach focused on the transformation of information (e.g., Cartesian space to joint torques; Kalaska and Crammond, 1992; Buneo et al., 2002; Todorov et al., 2005) or a modular approach where each level provides a distinct role (e.g., motor planning by M1, feedback control by subcortical circuits; Kawato et al., 1987; Schweighofer et al., 1998; Loeb et al., 1999; Merel et al., 2019). Alternatively, multiple levels may contribute to generating feedback responses, but without distinct roles captured by engineering principles. From this perspective, M1 contributes the extra motor ,nds necessary to attain a behavioral goal that is adjusted based on the contributions by lower levels. This could include a reduction in motor output when needed to compensate for increased contributions from lower circuits (e.g., gain scaling; Pruszynski et al., 2009). Unravelling the relative contributions of different levels of the motor system during voluntary control remains an important and challenging area of study.

## Synthesis

Reviewing Editor: Arvind Kumar, KTH Royal Institute of Technology Decisions are customarily a result of the Reviewing Editor and the peer reviewers coming together and discussing their recommendations until a consensus is reached. When revisions are invited, a fact-based synthesis statement explaining their decision and outlining what is needed to prepare a revision will be listed below. The following reviewer(s) agreed to reveal their identity: NONE.

Synthesis

Your manuscript was reviewed by two reviewers. Both thought that the question of how sensory and motor signals interact (or not) is important and the paper has the potential to add new data/ideas to the literature on this question.

But the reviewers have raised substantial concerns. Most importantly, the way it is presented in the manuscript, the experimental design does not seem to be appropriate for the question you want to address.

Both reviewers thought that the use of the first 300 ms window to construct different types of sensory subspaces is not appropriate. The activity in this interval also contains motor responses. So similarity of the responses to different sensory perturbations could be due to similarity of motor responses caused by those perturbations. Reviewers thought that this issue is critical to the paper and requires new analysis.

They have made suggestions how you could try to tease apart the sensory components from the motor ones e.g. by using a short analysis window or by comparing different motor response conditions. While new experiments may not be feasible, further analysis of the existing data could help settle some of the concerns of the reviewers.

In addition, the methods and particularly statistical analysis need more detailed description.

Comments from the two reviewers are appended below. These are all very important and should be addressed. Please provide a detailed point-by-point reply to these comments.

Summary Reviewer #1

This paper wants to test the independence/interaction of sensory inputs into primary motor cortex. This has been examined in previous work, some of which is cited (but see https://doi.org/10.1152/jn.1983.50.6.1343). The biggest issue I see here is that the way the hypothesis is framed is out of touch with the analysis itself. Critically, the use of a 300 ms window means that a lot of what is analyzed reflects the motor response itself as opposed to the sensory inputs per se. To me, this makes the paper very difficult to interpret because nothing can be linked to the sensory inputs directly. The choice of window interferes with essentially every analysis and the authors should use a much shorter and tighter window.

Summary Reviewer #2

The manuscript probes how M1 neurons respond to perturbations in vision and proprioception applied to the moving limb. As such, the work tackles the important issue of feedback control in sensorimotor neuroscience. The findings are interesting, and non-trivial.

It's very hard to tell. A lot of the analysis appears to factor out baseline within a condition. In general it seems important to keep that in across conditions. The state space analysis is very unclear esp since no graphical representation is provided.

The statistical analysis is ok, but can be significantly improved. Please see the detailed comments to the authors. Thanks.

Reviewer #1

This paper attempts to determine whether sensory inputs to M1 target the same/different neurons and/or neuronal subspaces. This is an interesting question and one that has been asked previously in a range of studies, some of which is cited (but see https://doi.org/10.1152/jn.1983.50.6.1343 which is very relevant and comes to different conclusions but is not mentioned).

From my perspective, there is one central issue that precludes any firm connection between the stated goal of the study and the analysis itself. The authors use a 300 ms window to quantify the sensory response but a lot of what this window contains will include the motor response itself as opposed to the sensory inputs per se. That is, it is entirely imaginable that early responses in this window are single modality but that later responses appear the same regardless of modality because the motor responses required are similar. The latter part of this is indeed what I think a simple model of M1 that produces muscle activity would predict. Luckily, this is something the authors can address by doing the analysis with a much tighter window around the earliest moment of the first sensory driven response (the earlier and tighter the better to get at the stated goal). Another interesting avenue then is to see how this evolves over time. This comment essentially applies to every analysis in the paper and so I don't go further into the details of these analyses at this point.

I will add that the analysis is very unclear. In particular how the background levels across conditions play into the ANOVAs. Also, the state space analysis is very difficult to parse and the visualizations are not helpful.

Reviewer #2

This paper probes the influence of sensory feedback on M1 neuron activity. Three types of perturbations across two different sensory modalities are applied during arm movement in two macaque monkeys - target jumps (vision), cursor jumps (vision) and mechanical loads (proprioception). The authors show strong overlap in M1 responses to the 3 different perturbations - both in terms of single neuron as well as population level activity. The authors conclude that sensory feedback from a variety of sensory modalities converges on the same M1 circuits.

In general, the experimental design is fine (but perhaps not the best to address the deeper question, see below) and the data analysis is thorough. I have some suggestions for the authors to consider:

1. It would be helpful to elaborate on whether and why motor tasks that involve perturbations to the moving limb are the best way to probe how sensory feedback influences M1. I am not convinced that this is the best paradigm to address the deeper issue, which is the goal of the authors. How would the finding of overlap in this case generalize to movements that do not involve perturbations? I am not sure these are the same in terms of M1 responses.

2. I think that what the authors are seeing may actually be related to the response required to compensate for the perturbation rather than the sensory feedback coming in. In all the results shown, the comparison is either across correction directions (collapsed), or across sensory modalities but within a correction direction. I suggest that the authors disambiguate this motor versus sensory issue. One way in which this could be done perhaps is to compare say a leftward correction to a target jump to a downward correction to a mechanical load. If overlap still exists, great. But if not, the implications of the findings will be completely different. The authors have the data, so this should be easily doable.

3. What about postural control? Are the authors suggesting overlap there as well?

4. I guess the larger point I am trying to make is that there needs to be a more convincing demonstration of overlap as a ubiquitous feature, and also that it is related to sensory feedback rather than the motor response. The paper makes sweeping statements implying both of these, but the evidence is limited to very few, narrow cases. At the very least, this issue should be discussed and tempered.

5. Some clarity could be brought in the Methods. I assume the visual feedback was blocked for 200 ms only in the case of the mechanical load. It may be explicitly mentioned then that this was not the case for the target jump and cursor jump trials.

6. The results section could benefit from a deeper analysis of the feedback / no-feedback trials. This is only fleetingly discussed, but is important. I don't think it is the case that these two conditions produce similar responses in M1 - clearly, removing cursor information, even briefly, led to a 33% increase in endpoint distance. It is also important to discuss how this impacts the overlap results.

7. I also think that the reporting of the results and the statistics could be much better. For example, on line 275, it is mentioned that the monkeys were able to initiate a correction within 200 ms. Likewise, on line 280, it is mentioned that one of the neurons showed a response to goal jumps within 100 ms. I think precise numbers should be provided, along with detailed statistics for all comparisons. Relatedly, please avoid use of the term "similar" in case of non-significant differences. The appropriate reporting term is "not different". This is an issue throughout the manuscript.

8. Please consider using Bayesian statistics, as is the general policy of this journal.

9. Line 267: "only 6% of muscle samples displayed significantly different....". What does this mean?

10. Lines 326-333: please show this statistically.

11. Please elaborate on why "response range" is the best measure for testing whether each sensory modality independently influences M1 by driving distinct activity patterns in the same neuron population.

12. Lines 366-372. Not sure how this paragraph fits with the rest of the discussion.

13. Please check the spelling of "principal" in the PCA section. There are many typos with "principle" being used.

## Author Response

We thank the reviewers for their feedback on our manuscript. Below is our point-by-point response to each comment in red bolded text. Italicized lettering referred to direct passage from the revised manuscript. Line numbers correspond to lines in the clean version of the manuscript.

Reviewer #1

The authors provide a revised manuscript that addresses at some level the issues raised by the reviewers. I am still left unsatisfied at the end of the paper, specifically about how it is framed given the experiment and results -- not that the results are wrong per se but rather that it is really hard to understand the main message. Would either of the two hypotheses (independence, convergence) lead to a conclusion that M1 circuits are highly organized? In fact, the answer lies somewhere in between the hypotheses but this is also considered organized? I think the authors owe it to the reader to lay out what they mean here if they are leading with such a dichotomous hypothesis or perhaps it is just best to make this all much more descriptive and then more carefully report the nature of the responses. What do I learn about M1 specifically? More so since there's many conflicting prior results in related paradigms as the authors mention.

We think the two extreme hypotheses laid out in the Introduction are important to provide the reader with two possible outcomes for our experiment. However, our choice of literature to motivate these hypotheses could have been better selected. We have now focused more on previous neurophysiological examinations of M1 and less on the previous behavioural studies. For the independent input hypothesis, we have now focused the Introduction on previous studies of cutaneous and proprioceptive feedback responses in M1 and how they appear to target distinct pools of neurons in M1. Further, we draw on several anatomical studies showing how different areas target distinct circuits in M1 with areas carrying more visual signals targeting more rostral M1 and areas carrying more proprioceptive signals targeting more caudal M1. For the convergence hypothesis, support is more limited and based on computational frameworks from the literature as well as a previous study examining M1's responses to movement triggered by different sensory modalities (Lamarre et al. 1983). It is worthing noting that the amount of literature on multi-modal responses in M1 is rather shallow highlighting an important need for the present study. The Introduction now reads (starting line 86) "On one extreme, each feedback source may influence M1 independently to generate the motor correction (independence hypothesis). The motor system rapidly responds to proprioceptive and visual feedback, which may not allow the brain sufficient time to perform the necessary non-linear computations. Anatomical evidence highlights how different areas involved with sensory processing appear to target spatially distinct circuits in M1. For example, somatosensory and parietal areas project to more caudal subdivisions of M1 whereas areas involved with visual processing such as premotor cortex project to more rostral subdivisions (Dea et al., 2016). Further, many studies highlight how M1 neurons that responded to cutaneous and proprioceptive inputs are distinct with only rare instances of neurons that responded to both stimuli (Rosén and Asanuma, 1972; Lemon et al., 1976; Wong et al., 1978; Fetz et al., 1980; Tanji and Wise, 1981; Picard and Smith, 1992) . These studies suggest that inputs carrying sensory information to M1 may be segregated leading to recruitment of distinct circuits in M1 for proprioceptive and visual corrective motor responses with overlap only by random chance.

Alternatively, feedback sources could recruit a similar population of M1 neurons to generate the motor correction (convergence hypothesis). Integrated feedback is an assumption in many control schemes where a difference vector is computed between the location of the goal and hand, which is then converted to motor commands (Bullock et al., 1998; Sober and Sabes, 2003; Shadmehr and Wise, 2005; Burns and Blohm, 2010). A common assumption is the computation of the difference vector is occurs in premotor and parietal cortices where neural signatures of this computation have been found (Buneo et al., 2002; Pesaran et al., 2006; McGuire and Sabes, 2011; Bremner and Andersen, 2012; Piserchia et al., 2017). Previous studies have also shown that monkeys trained to initiate a reaching movement by different sensory triggers (e.g. auditory tone, visual cue) recruit the same M1 neuron population (Lamarre et al., 1983). The prediction is that a common group of neurons in M1 should respond to mechanical and visual disturbances of the limb as well as visual disturbances of the goal."

As the reviewer correctly identifies, our study findings lie somewhere in the middle of these two hypotheses. For the two visual perturbations appear more strongly biased towards the convergence hypothesis. In contrast, comparing the responses to the mechanical perturbation with either visual perturbation are more in the middle of the two hypotheses. We have now clearly stated this in the Discussion which now reads (starting line 515) "We set out to identify how different sensory sources recruit M1 circuits. Our initial hypotheses considered two extreme possibilities, 1) different sensory errors recruit different circuits in M1 (independent hypothesis) or 2) different sensory errors recruit the same circuits in M1 (converge hypothesis). Our findings highlight how visual feedback of the limb and visual feedback of the goal evoke highly similar patterns of M1 activity more consistent with the convergence hypothesis. Examination of the population-level analysis (Figure 9 and 10) highlights how goal and cursor jumps evoke activity patterns in subspaces that were highly similar and differed only slightly more than comparing trials from the same perturbation type (i.e. the within-perturbation). At the single-neuron level, we found a large group of neurons that responded to both the cursor and goal jumps (Figure 7) and exhibited similar activity patterns (Figure 6).

However, it is worth noting that there was a substantive population of neurons that had significant responses to only one of the visual perturbations (Figure 6B, D). It is interesting that despite these distinct populations of neurons that only a small difference was detected in the low-dimensional neural subspace. This may indicate that population-level analysis may miss important characteristics found at the single-neuron level. Alternatively, unique neuron populations may be an artifact in part caused by binary classification of neurons using an ANOVA approach.

A clearer divergence from the two extreme hypotheses is highlighted when contrasting perturbations of vision and proprioception. At the single-neuron level proprioceptive and visual perturbations modulated overlapping populations of neurons with ~43% of perturbation-sensitive neurons responding to all three perturbations which is considerably more than random chance (~16%). This overlap was also observed at the population-level where each perturbation evoked activity that occupied overlapping neural subspaces. However, the overlap was considerably weaker than the cursor- and goal-jump comparison. It is possible that the weaker overlap may in part reflect differences in the required motor output as muscle activity evoked by visual and proprioceptive perturbations were not as strongly overlapped as the overlap between the cursor- and goal-jump muscle activity patterns (Figure 9I-L). As such these results may reflect a lower bound on the convergence of visual and proprioceptive feedback responses on M1 neurons. Collectively, these results suggest a partial overlap between proprioceptive and visual responses thus residing in the middle of the convergent input hypothesis and independent input hypothesis."

Finally, we think these feedback responses show a level of organization as our analysis routinely shows the distribution of feedback responses across M1 neurons are stronger than random chance. However, to be more concise throughout the manuscript we have elected to remove reference to an organization of M1 responses.

#Reviewer 3

I believe that the authors have made substantial changes to the manuscript in response to the major concerns I had. The newer analyses examining the response ranges near the onset of the perturbation are also helpful. They reveal trends similar to the response range analyses over a much larger time window that could potentially include the motor response. However, I cant help but feel that this in fact strengthens the concern that both Reviewer 1 and I had pointed about the overlapping neural activity being related to the motor response rather than the sensory input. The authors subtly acknowledge this, but it would be helpful if this were made crystal clear in the manuscript. I don't have any other serious concerns.

Please see response to Reviewer 1. Also, we agree with the reviewer that the evoked responses to the different sensory perturbations likely reflect a combination of both sensory and motor responses that are overlapped. We have made this more clearly with two lines in the manuscript. First in the Results section when introducing the perturbation epoch, we include the following (line 408-409) "Note that this epoch likely reflects a combination of both sensory and motor processing."

Also, at the beginning of the Discussion we mention (starting line 579-581) "Our results indicate sensory perturbations recruited a rapid and robust response in M1 activity which likely reflects both sensory processing and the corrective motor commands."
