# Supplementary material for: Rapid Online Corrections for Proprioceptive and Visual Perturbations Recruit Similar Circuits in Primary Motor Cortex
Source: eNeuro. 2024 Feb 9;11(2):ENEURO.0083-23.2024. doi: 10.1523/ENEURO.0083-23.2024 (PMC10867723; doi:10.1523/ENEURO.0083-23.2024)
Supplement: Table 1. — Onsets for M1 neurons identified as responsive to target-only, cursor-only and mechanical-only perturbations. Neurons were pooled across both monkeys. Obs: observed, Exp: expected. Download Table 1, DOCX file. [file eneuro-11-ENEURO.0083-23.2024-s002.doc]

| Extended Table 1: Onsets for M1 neurons identified as responsive to target-only, cursor-only and mechanical-only perturbations. Neurons were pooled across both monkeys. Obs: observed, Exp: expected. | | | | | | | | |
| --- | --- | --- | --- | --- | --- | --- | --- | --- |
|  |  | 25-50ms | 50-75ms | 75-100ms | 100-125ms | 125-150ms | 150-175ms | 175-200ms |
| Target-Only | Obs. | 0 | 0 | 1 | 1 | 2 | 4 | 0 |
| N=8, χ^2^=2.4 | Exp. | 0.08 | 0 | 1.18 | 2.02 | 2.02 | 2.27 | 0.42 |
|  |  |  |  |  |  |  |  |  |
| Cursor-Only | Obs. | 0 | 0 | 1 | 0 | 0 | 0 | 0 |
| N=1, χ^2^=undef | Exp. | 0 | 0.01 | 0.05 | 0.17 | 0.2 | 0.16 | 0.15 |
|  |  |  |  |  |  |  |  |  |
| Mech-Only | Obs. | 0 | 5 | 3 | 3 | 1 | 0 | 1 |
| N=13, χ^2^=4.1 | Exp. | 0.14 | 2.87 | 4.93 | 1.92 | 1.64 | 0.68 | 0.82 |
