# Supplementary figures and images for: Rapid Online Corrections for Proprioceptive and Visual Perturbations Recruit Similar Circuits in Primary Motor Cortex
Source: eNeuro. 2024 Feb 9;11(2):ENEURO.0083-23.2024. doi: 10.1523/ENEURO.0083-23.2024 (PMC10867723; doi:10.1523/ENEURO.0083-23.2024)

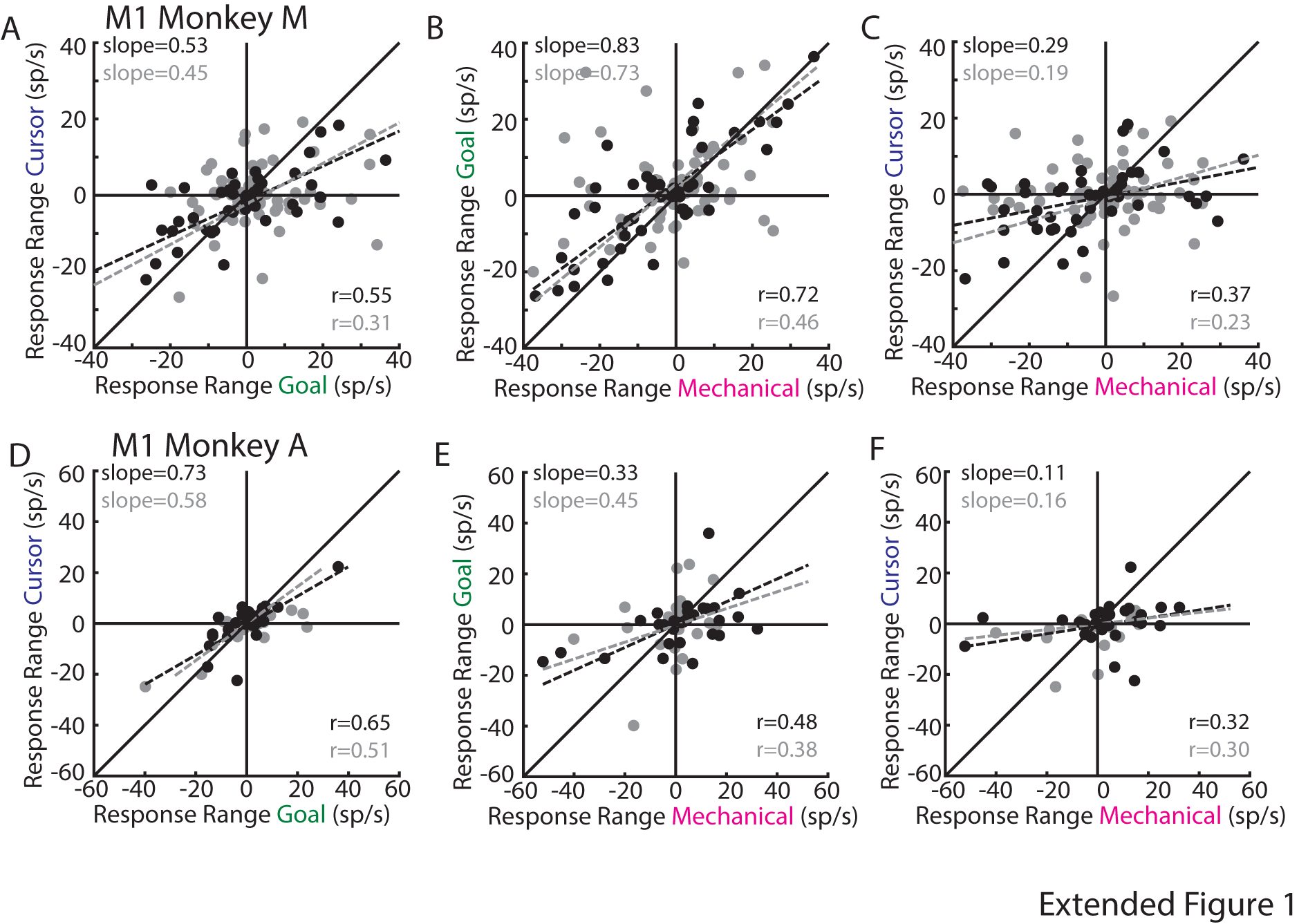

Supplement: Figure 1. — M1 neurons have similar response ranges across perturbation types during the onset of evoked activity. Data are presented the same as Figure 7 except response ranges were calculated for the visual perturbations from 75-125ms after perturbation onset and for the mechanical perturbations from 50-100ms after the perturbation onsets. Download Figure 1, TIF file. [file eneuro-11-ENEURO.0083-23.2024-s001.tif]
